# Equations for estimating ileal amino acid digestibility and digestible energy in bakery byproducts fed to nursery pigs

**DOI:** 10.5713/ab.251021

**Published:** 2026-03-11

**Authors:** Hee Eun Oh, Yoon Soo Song, Hyunseok Do, Yeojin An, Beob Gyun Kim

**Affiliations:** 1 Department of Animal Science, Konkuk University, Seoul, Korea; 2 Monogastric Animal Feed Research Institute, Konkuk University, Seoul, Korea

**Keywords:** Amino Acid, Bakery Meal, Energy Digestibility, Prediction Models, Swine

## Abstract

**Objective:**

The present experiment aimed to determine standardized ileal digestibility (SID) of amino acids (AA) and digestible energy (DE) in four bakery byproducts fed to nursery piglets and to develop prediction equations for estimating SID of AA and DE in bakery byproducts.

**Methods:**

Five barrows with an initial body weight of 16.6 kg (standard deviation = 1.7) were fitted with a T-cannula at the distal ileum and individually housed in pens. A 5×5 Latin square design was used with 5 treatments, 5 pigs, and 5 periods. Four sources of bakery byproducts containing 9.1% to 38.4% neutral detergent fiber (NDF) were used. Three experimental diets contained each bakery byproduct at 96.6% as the sole source of nitrogen and energy. The fourth experimental diet consisted of a source of bakery byproduct (38.4% NDF) at 70.0% as the sole source of nitrogen. A nitrogen-free diet was prepared to determine the basal endogenous AA losses. Each period consisted of 6 d of adaptation, 1 d of fecal collection, and 3 d of ileal collection.

**Results:**

The SID of most AA and DE differed (p<0.05) among 4 bakery byproducts. The NDF concentration was negatively correlated (p<0.05) with the SID of indispensable AA except Lys and DE in bakery byproducts. Prediction equations were developed for estimating SID of AA and DE: SID of Met (%) = 86.7–0.54×NDF (r^2^ = 0.74 and p<0.001); SID of Thr (%) = 73.4–1.00×NDF (r^2^ = 0.57 and p<0.001); and DE (kcal/kg as-is) = 4,819–81.96×NDF (r^2^ = 0.93 and p<0.001), where NDF is expressed as % as-is.

**Conclusion:**

Amino acid digestibility and DE variede among bakery byproducts fed to nursery piglets. Neutral detergent fiber can be used to estimate SID of AA and DE in bakery byproducts for nursery piglets.

## INTRODUCTION

Grains such as corn and wheat are the major feed ingredients incorporated in swine diets mainly for providing energy [1]. As the demand for grains is increasing due to the growing human population, the competition for grains as animal feed and human food is intensifying, potentially raising the grain prices and the cost of pork production [2–4]. Food wastes are considered a sustainable alternative feed ingredient that could potentially replace conventional feed ingredients in swine diets, among which bakery byproducts, typically consisting of breads, cakes, snacks, pasta, and flours, have gained attention and are often used in swine diets due to the high sugar and starch concentrations [5,6]. The energy values and the amino acid (AA) digestibility have been reported to be relatively consistent among bakery byproducts sourced from different regions in the United States when fed to pigs [7,8]. However, nutrient compositions are largely variable among the bakery byproduct sources commercially available as a feed ingredient [9].

Different raw materials for producing bakery foods may cause nutritional variability in bakery byproducts, and fibrous materials are sometimes added for producing bakery byproducts as feed ingredients for animals [5,9]. The variability in nutrient composition likely affects nutrient digestibility as demonstrated in an *in vitro* study [9]. However, *in vivo* data are not available on the variability in AA and energy digestibility of bakery byproducts in nursery pigs. The objectives of the present experiment, therefore, were to determine standardized ileal digestibility (SID) of AA and apparent total tract digestibility (ATTD) of gross energy (GE) in various sources of bakery byproducts fed to nursery pigs and to develop prediction models for estimating SID of AA and digestible energy (DE) in bakery byproducts. The hypothesis was that the digestibility of AA and energy in bakery byproducts would change depending on the nutrient composition, particularly the fiber concentration.

## MATERIALS AND METHODS

### Animals, experimental design, and diets

Five barrows (Landrace×Yorkshire) with an initial body weight of 16.6 kg (standard deviation = 1.7) fitted with a T-cannula at the distal ileum were individually housed in pens of 1.2 m×1.6 m that were equipped with a feeder and a nipple drinker. The cannulation procedure was adapted from Stein et al [10]. Following surgery, the cannulas and wounds were disinfected daily. To support recovery, pigs were treated with an antibiotic (Buta-D PPS; DAESUNG Microbiological Labs) every 3 d for 2 wk. After the recovery period, the animals were allotted to 5 experimental diets in a 5×5 Latin square design for 5 periods to obtain 5 observations for each treatment.

Four sources of bakery byproducts with a wide range of amylase-treated neutral detergent fiber (NDF) concentration (9.1% to 38.4%) were obtained from commercial feed mills in Korea (Table 1). Three experimental diets were prepared to contain each bakery byproduct (A, B, or C) at 96.6% as the sole source of nitrogen and energy (Tables 2 and 3). The fourth experimental diet consisted of a source of bakery byproduct (D) containing 38.4% NDF at 70.0% as the sole source of nitrogen. The inclusion rate of the bakery byproduct-D was based on a preliminary palatability test. A nitrogen-free diet was prepared to determine the basal endogenous losses (BEL) of AA. In the diet for bakery byproduct-D and the nitrogen-free diet, the proportions of cornstarch, sucrose, soybean oil, and cellulose were identical, which enabled the calculation of energy values in the bakery byproduct-D using the difference procedure. Vitamins and minerals were included to meet or exceed the requirement estimates [11]. Chromic oxide was used at 0.5% as an indigestible index.

### Feeding and sample collection

Daily feed allowance was approximately 5% of body weight of each pig. The quantity of daily feed allowance was adjusted at the beginning of each period based on the body weight. The daily feed allowance was divided into three equal meals and fed to pigs at 09:00, 13:00, and 17:00 h. Water was available at all times. An experimental period consisted of 6 d of adaptation, 1 d of fecal collection, and 3 d of ileal digesta collection [12]. After the adaptation period, feces were collected for 24 h from 09:00 h on d 7. Ileal digesta samples were collected from 09:30 to 17:00 h on d 8, 9, and 10. A plastic sample bag with a wire was fixed to the T-cannula to collect the ileal digesta. Collected feces and ileal digesta were immediately stored at −20°C.

### Chemical analyses

The frozen feces were dried in a forced-air drying oven at 55°C until constant weight was achieved, and the frozen ileal digesta were dried in a vacuum freeze dryer (SFDTS10K; Samwon Freezing Engineering). The ingredients, diets, and feces were analyzed for GE using bomb calorimetry (Parr 6400; Parr Instruments). All chemical analyses were performed according to the AOAC International [13]. Dry matter (DM; method 930.15), crude protein (CP; method 990.03), ether extract (method 920.39), ash (method 942.05), amylase-treated NDF (method 2002.04), and acid detergent fiber (method 973.18) in ingredients and diets were determined. Ileal digesta were also analyzed for DM and CP. Amino acids in ingredients, diets, and ileal digesta were determined through ion-exchange chromatography with post-column derivatization using ninhydrin. Before AA analyses, samples were hydrolyzed using 6 mol/L HCl for 24 h at 110°C (method 982.30 E[a]). Sulfur-containing AA were analyzed as methionine sulfone and cysteic acid following cold performic acid oxidation overnight before digestion by HCl (method 982.30 E[b]). Tryptophan was analyzed after hydrolysis with NaOH for 22 h at 110°C (method 982.30 E[c]]. Chromium (Cr) in diets, ileal digesta, and feces were analyzed using a UV/Vis spectrophotometer (Optizen 2120UV; Mecasys).

### Calculations

The apparent ileal digestibility (AID) of AA and ATTD of GE in diets were calculated using the following Eq. 1 [14]:


(1) $$\text{AID or ATTD }(\%)=100-({\text{Nutr}}_{\text{digesta}}/{\text{Nutr}}_{\text{diet}})\times ({\text{Cr}}_{\text{diet}}/{\text{Cr}}_{\text{digesta}})\times 100$$

where Nutr_digesta_ is the AA concentrations (g/kg) in ileal digesta or GE (kcal/kg) in feces, Nutr_diet_ is the AA concentrations (g/kg) or GE (kcal/kg) in the diets, Cr_diet_ is the Cr concentrations (g/kg) in the diets, and Cr_digesta_ is the Cr concentrations (g/kg) in ileal digesta or feces.

The BEL of CP and AA (g/kg DM intake) were estimated using the prediction equations reported by Park et al [15] as the values determined in the present experiment were relatively high. The SID of CP and AA was calculated based on the following Eq. 2 [14]:


(2) $$\text{SID }(\%)=\text{AID }(\%)+(100\times [\text{BEL}/{\text{AA}}_{\text{diet}}])$$

where AA_diet_ is the CP or AA concentrations (g/kg DM) in the diets. The ATTD of GE in bakery byproduct-D was calculated using the following Eq. 3 [16]:


(3) $$\;{\text{ATTD}}_{\text{ingredient}}\;(\%)=({\text{ATTD}}_{\text{diet}}\;[\%]-\{{\text{ATTD}}_{\text{basal}}\;[\%]\times {\text{PC}}_{\text{basal}}\})/{\text{PC}}_{\text{ingredient}}$$

where ATTD_ingredient_ is the ATTD of GE in bakery byproduct-D, ATTD_diet_ is the ATTD of GE in the diet containing bakery byproduct-D, ATTD_basal_ is the ATTD of GE in the nitrogen-free diet, PC_basal_ is the proportional contribution of GE by cornstarch, sucrose, soybean oil, and cellulose to the bakery byproduct-D diet, and PC_ingredient_ is the proportional contribution of GE by bakery byproduct-D to the bakery byproduct-D diet. Digestible energy in bakery byproducts was calculated using the following Eq. 4:


(4) DE (kcal/kg)=GE (kcal/kg)×ATTD of GE (%)

Metabolizable energy (ME) in bakery byproducts was estimated using the prediction equation reported in the literature [17]:


(5) ME (kcal/kg)=DE (kcal/kg)-0.68×CP (g/kg)

### Statistical analyses

Data were analyzed using the MIXED procedure of SAS (SAS Institute). Outliers were identified if the values deviated by more than 1.5 times the interquartile range from the 1st or 3rd quartiles, and one observation from a pig fed the bakery byproduct-D diet was detected as an outlier. The model included dietary treatment as a fixed variable and animal and period as random variables. Least squares means were calculated for each treatment, and the means were separated using the PDIFF option with Tukey’s adjustment. Correlation coefficients among chemical composition and digestibility were determined by the CORR procedure of SAS. Prediction equations for SID of AA, ATTD of GE, and DE in bakery byproducts were developed via the REG procedure using NDF concentration as an independent variable. The experimental unit was a pig, and the statistical significance was declared at p<0.05.

## RESULTS

The AID of CP and all AA differed (p<0.05) among bakery byproducts with the lowest AID of CP and most AA in bakery byproduct-D (Table 4). The SID of CP and all AA except Trp and Pro differed (p<0.05) among bakery byproducts with the lowest SID of most AA in bakery byproduct-D (Table 5).

The ATTD of GE in bakery byproduct-A and -B was greater (p<0.05) than that in bakery byproduct-C and bakery byproduct-D (Table 6). The DE and the calculated ME in bakery byproduct-A and bakery byproduct-B were greater (p<0.05) than those in bakery byproduct-C and bakery byproduct-D.

The NDF concentration in bakery byproducts was negatively correlated with the SID of CP (r = −0.718 and p<0.001), Met (r = −0.859 and p<0.001), Thr (r = −0.758 and p<0.001), and Trp (r = −0.516 and p<0.05; Table 7). In addition, the NDF concentration was negatively correlated with the ATTD of GE (r = −0.944 and p<0.001) and DE (r = −0.965 and p<0.001) in bakery byproducts as well.

The prediction equations for estimating SID of CP and AA developed were: SID of CP (%) = 86.6–0.78×NDF with r^2^ = 0.52 and p = 0.001; SID of Met (%) = 86.7–0.54×NDF with r^2^ = 0.74 and p<0.001; SID of Thr (%) = 73.4–1.00×NDF with r^2^ = 0.57 and p<0.001 (Table 8). The prediction equation for estimating DE was: DE (kcal/kg as-is) = 4,819–81.96×NDF (r^2^ = 0.93 and p<0.001; Table 9). The NDF concentration is expressed as % on an as-is basis.

## DISCUSSION

Bakery byproducts have been highlighted in the swine feed industry due to their similar or greater energy values compared with grains and their excellent palatability for pigs [18,19]. Previously, pigs fed a diet containing bakery byproducts to replace grains showed similar feed efficiency and nutrient digestibility and a positive change in fecal microbiota compared with the control group [6]. However, bakery byproducts are potentially variable in nutrient composition depending on the quality of the food materials. In addition, high-fiber ingredients such as almond hulls and rice bran are often incorporated into bakery byproducts as excipients or desiccants, increasing fiber concentration, reducing other nutrient concentrations, and potentially altering nutrient digestibility in pigs [9]. In a previous *in vitro* study, the NDF concentrations in 10 sources of bakery byproduct ranged from 15% to 50% as-is and were strongly negatively correlated with digestibility of DM and CP [9]. Therefore, bakery byproducts containing a wide range of NDF concentrations were used to evaluate the digestibility of AA and energy in pigs.

The analyzed concentrations of CP, ash, and most AA in the 4 bakery byproducts used in the present experiment were within the range of previously reported values [5,7–9]. Bakery byproduct-A contained a greater GE concentration compared with other sources used in the present and previous experiments primarily due to the high fat concentration [20]. Bakery byproduct-A may have consisted mainly of high-fat food materials such as high-oil desserts. The NDF concentrations in bakery byproduct-C and -D were higher than those in the other sources used in the present and previous experiments [5,18,21], which may have resulted from the inclusion of high-fiber food materials or excipients, but remained within the range of data reported by Oh et al [9].

In the present experiment, the very low AID values for Gly and Pro in bakery byproducts were observed likely due to the BEL of Gly and Pro derived from sloughed epithelial cells [15,22]. In addition, bile salts rich in Gly and mucin rich in Pro are highly resistant to proteolysis in the intestine, which further contributes to the substantial amount of Gly and Pro losses [15,22]. The large amounts of BEL of AA result in underestimation of AA digestibility in pigs [15,23,24]. In addition, dietary fiber can increase ingredient-specific endogenous losses, including AA-rich sloughed epithelial cells, mucin, and digestive secretions [25]. Although only BEL of AA was used to calculate the SID of AA in the ingredients, ingredient-specific endogenous losses of AA for bakery byproduct-D would be greater than those for other bakery byproducts due to the greater fiber concentration in bakery byproduct-D.

The SID of most AA was less in bakery byproduct-D compared with the other sources, which is primarily attributed to the high fiber concentration in bakery byproduct-D. Ingested fibers can disturb the digestion and absorption of nutrients by encapsulating the nutrients in the digesta [26,27]. In addition, high dietary fiber concentration increases digesta volume and stimulates gastrointestinal motility, resulting in a faster passage rate and reduced time for nutrient digestion and absorption [28]. These mechanisms at least partially explain the negative correlations between NDF and the SID of most AA in bakery byproducts observed in the present work, which is consistent with the previous experiment that observed a negative correlation between NDF and CP digestibility [9].

Although the SID of most indispensable AA in bakery byproduct-B was the greatest among bakery byproducts due to the low fiber concentration, the SID of Lys was the lowest, resulting in no correlation between NDF and the SID of Lys, which may be attributed to the Maillard reaction. In the presence of moisture and heat, the epsilon amino group of Lys easily reacts with reducing sugars, forming Amadori compounds and melanoidins [29–31]. Therefore, excessive heat treatments during food processing may have led to a decrease in Lys concentration and digestibility in foods [8]. In this regard, bakery byproduct-B may consist of more heat-damaged food materials compared with other bakery byproducts, resulting in the low Lys-to-CP ratio and low Lys digestibility.

The ATTD of GE in bakery byproduct-A and bakery byproduct-B was within the range of values reported in previous studies [7,21] likely due to the comparable nutrient composition, particularly the fiber concentrations. In contrast, the ATTD of GE in bakery byproduct-C and bakery byproduct-D was less than that in bakery byproduct-A and bakery byproduct-B mainly due to the higher fiber concentrations, resulting in a strong negative correlation between NDF and ATTD of GE observed in the present experiment. Similarly, Oh et al [9] observed a negative correlation between NDF and the ATTD of DM and organic matter, which further supports the present observations. The negative correlation between NDF concentration and nutrient digestibility is attributed to the fact that dietary fiber is less digestible than other nutrients and disrupts the action of digestive enzymes on substrates in the gastrointestinal tract [32–34].

Previous experiments suggested that the nutrient compositions and availability in bakery byproducts sourced from different regions in the United States showed a relatively narrow range compared with other feed ingredients for pigs [5,7,8]. Within this range of nutrient compositions, bakery byproduct-A and bakery byproduct-B had comparable digestible AA concentrations and greater energy values, thus showing the potential to replace grains in swine diets. Previous experiments also reported that growth performance in pigs was not affected by incorporating bakery byproducts to partially replace corn in swine diets [6,35]. In contrast, bakery byproducts may contain high fiber concentrations like bakery byproduct-C and bakery byproduct-D which are less digestible in AA and energy compared with some grains. Apparently, fiber contents can be used for estimating DE in bakery byproducts and potentially for estimating SID of AA when formulating nursery pig diets. The NDF-based prediction equations proposed in the present study allow estimation of AA and energy digestibility in bakery byproducts without the need for animal experiments. The prediction equations were developed using bakery byproducts with NDF concentrations ranging from 9.1% to 38.4%. Therefore, caution is needed when applying these equations to bakery byproducts with NDF concentrations outside this range. Furthermore, given the wide variation in NDF concentrations among bakery byproducts, additional study evaluating the growth performance of nursery pigs fed diets that partially replace grains with high-fiber bakery byproducts is needed.

## CONCLUSION

The SID of AA and the ATTD of GE differed among the various bakery byproducts due to the different nutrient compositions in nursery pigs. Based on the strong correlations observed, NDF was used to estimate the SID of AA and DE concentrations in bakery byproducts for nursery pigs. By using the prediction models, digestible AA and DE in bakery byproducts with different fiber concentrations can be applied in swine diet formulations. However, the prediction models were developed using a limited number of samples. Therefore, further studies are warranted to validate the prediction models using independent datasets, investigate additional chemical predictors, and evaluate the effects of bakery byproducts on growth performance of nursery pigs.

## Figures and Tables

**Table 1:** Energy and nutrient composition of bakery byproducts (as-is basis)

Item	Bakery byproduct-A	Bakery byproduct-B	Bakery byproduct-C	Bakery byproduct-D
Dry matter (%)	93.87	91.68	86.93	87.45
Gross energy (kcal/kg)	5,194	4,523	4,308	4,037
Crude protein (%)	8.22	11.88	10.93	9.51
Ether extract (%)	26.62	11.67	12.45	8.83
Ash (%)	3.89	2.32	5.71	10.04
Neutral detergent fiber (%)	9.07	12.65	18.88	38.42
Acid detergent fiber (%)	6.14	8.12	11.30	26.75
Indispensable amino acids (%)				
Arg	0.67	0.63	0.77	0.74
His	0.26	0.33	0.37	0.29
Ile	0.44	0.51	0.53	0.54
Leu	0.58	1.00	0.85	0.71
Lys	0.37	0.26	0.51	0.40
Met	0.15	0.21	0.21	0.19
Phe	0.30	0.58	0.50	0.39
Thr	0.34	0.47	0.48	0.42
Trp	0.06	0.10	0.09	0.06
Val	0.29	0.61	0.60	0.41
Dispensable amino acids (%)				
Ala	0.33	0.39	0.64	0.48
Asp	1.09	0.77	1.00	0.80
Cys	0.15	0.23	0.22	0.20
Glu	2.06	3.48	2.40	1.96
Gly	0.39	0.49	0.58	0.48
Pro	0.49	1.10	0.78	0.69
Ser	0.42	0.72	0.63	0.56
Tyr	0.15	0.24	0.24	0.13

**Table 2:** Ingredient composition of experimental diets

Item (%)	Diets containing bakery byproducts				Nitrogen-free diet

	A	B	C	D	
Bakery byproduct-A	96.6	-	-	-	-
Bakery byproduct-B	-	96.6	-	-	-
Bakery byproduct-C	-	-	96.6	-	-
Bakery byproduct-D	-	-	-	70.0	-
Cornstarch	-	-	-	17.7	63.5
Sucrose	-	-	-	6.97	25.0
Soybean oil	-	-	-	0.84	3.00
Cellulose	-	-	-	1.12	4.00
Potassium carbonate	-	-	-	-	0.40
Magnesium oxide	-	-	-	-	0.10
Ground limestone	0.75	0.75	0.75	0.42	0.43
Dicalcium phosphate	1.23	1.23	1.23	2.00	2.16
Sodium chloride	0.40	0.40	0.40	0.40	0.40
Vitamin-mineral premix^1)^	0.50	0.50	0.50	0.50	0.50
Chromic oxide	0.50	0.50	0.50	0.50	0.50

1) Provided the following quantities per kilogram of complete diet: vitamin A, 30,000 IU; vitamin D_3_, 6,000 IU; vitamin E, 100 IU; vitamin K, 7.5 mg; thiamin, 7.5 mg; riboflavin, 12.5 mg; pyridoxine, 7.5 mg; vitamin B_12_, 0.1 mg; pantothenic acid, 50 mg; folic acid, 2.5 mg; niacin, 75 mg; biotin, 0.5 mg; Co, 1.25 mg as cobaltous sulfate; Cu, 100 mg as copper sulfate; Fe, 200 mg as iron sulfate; I, 200 mg as calcium iodate; Mn, 100 mg as manganese sulfate; Se, 0.5 mg as sodium selenite; and Zn, 100 mg as zinc sulfate.

**Table 3:** Analyzed composition of experimental diets (as-fed basis)

Item	Diets containing bakery byproducts				Nitrogen-free diet

	A	B	C	D	
Dry matter (%)	93.87	91.56	87.09	88.35	92.06
Gross energy (kcal/kg)	5,053	4,381	4,158	3,869	3,715
Crude protein (%)	8.05	11.57	10.76	6.86	0.52
Ether extract (%)	25.39	11.29	11.53	7.14	3.22
Ash (%)	6.43	4.73	8.08	10.04	3.67
Neutral detergent fiber (%)	8.98	11.4	18.42	27.04	4.36
Acid detergent fiber (%)	5.82	6.15	10.90	19.08	3.91
Indispensable amino acids (%)					
Arg	0.70	0.63	0.84	0.61	-
His	0.25	0.33	0.34	0.24	-
Ile	0.43	0.63	0.52	0.44	-
Leu	0.58	1.00	0.75	0.58	-
Lys	0.36	0.27	0.45	0.31	-
Met	0.14	0.20	0.19	0.16	-
Phe	0.30	0.58	0.43	0.27	-
Thr	0.33	0.47	0.42	0.31	-
Trp	0.05	0.12	0.09	0.05	-
Val	0.30	0.59	0.56	0.28	-
Dispensable amino acids (%)					
Ala	0.32	0.46	0.51	0.34	-
Asp	1.02	0.77	0.85	0.60	-
Cys	0.15	0.21	0.21	0.16	-
Glu	1.93	3.48	2.08	1.40	-
Gly	0.39	0.49	0.52	0.35	-
Pro	0.56	0.90	0.62	0.52	-
Ser	0.41	0.72	0.53	0.38	-
Tyr	0.17	0.24	0.21	0.08	-

**Table 4:** Apparent ileal digestibility (%) of crude protein and amino acids in bakery byproducts fed to nursery pigs

Item	Bakery byproduct-A	Bakery byproduct-B	Bakery byproduct-C	Bakery byproduct-D	SEM	p-value
No. of observations	5	5	5	4		
Crude protein	57.4^a^	59.5^a^	59.5^a^	32.2^b^	4.0	0.001
Indispensable amino acids						
Arg	72.3^a^	66.3^a^	68.3^a^	39.0^b^	2.8	<0.001
His	60.8^a^	66.3^a^	58.4^a^	34.6^b^	2.8	<0.001
Ile	66.7^b^	75.0^a^	62.0^b^	47.6^c^	2.1	<0.001
Leu	66.8^ab^	75.5^a^	61.6^bc^	52.5^c^	3.2	0.002
Lys	62.5^a^	26.7^b^	55.1^a^	35.8^b^	3.4	<0.001
Met	74.0^a^	77.2^a^	71.4^a^	60.4^b^	1.7	<0.001
Phe	61.8^b^	79.1^a^	58.8^b^	30.9^c^	3.8	<0.001
Thr	44.0^a^	53.3^a^	41.5^a^	17.4^b^	4.6	0.002
Trp	49.5^ab^	74.8^a^	51.2^ab^	27.8^b^	8.6	0.012
Val	52.4^a^	65.6^a^	59.5^a^	27.7^b^	5.4	0.001
Dispensable amino acids						
Ala	47.0^a^	49.7^a^	51.9^a^	21.7^b^	3.6	0.001
Asp	72.7^a^	52.0^b^	52.8^b^	33.3^c^	3.0	<0.001
Cys	61.7^a^	65.7^a^	59.2^a^	38.6^b^	2.3	<0.001
Glu	81.2^ab^	84.9^a^	75.4^b^	62.0^c^	1.6	<0.001
Gly	23.5^a^	22.7^a^	27.3^a^	−32.0^b^	10.7	0.004
Pro	−85.3^ab^	−43.9^a^	−79.5^ab^	−217.4^b^	47.2	0.026
Ser	54.2^a^	64.8^a^	51.0^a^	24.7^b^	3.7	<0.001
Tyr	34.4^a^	54.7^a^	33.0^a^	−79.6^b^	10.1	<0.001

a–c Least squares means without a common superscript letter differ (p<0.05).

SEM, standard error of the means.

**Table 5:** Standardized ileal digestibility (%) of crude protein and amino acids in bakery byproducts fed to nursery pigs

Item	Bakery byproduct-A	Bakery byproduct-B	Bakery byproduct-C	Bakery byproduct-D	SEM	p-value
No. of observations	5	5	5	4		
Crude protein	79.6^a^	74.5^a^	74.9^a^	56.8^b^	4.0	0.006
Indispensable amino acids						
Arg	81.6^a^	76.3^a^	75.4^a^	49.0^b^	2.8	<0.001
His	68.6^a^	72.2^a^	63.8^a^	42.3^b^	2.8	<0.001
Ile	74.3^ab^	80.1^a^	67.9^b^	54.6^c^	2.1	<0.001
Leu	76.1^ab^	80.7^a^	68.2^bc^	61.1^c^	3.2	0.004
Lys	74.9^a^	42.8^b^	64.2^a^	49.4^b^	3.4	<0.001
Met	80.6^a^	81.8^a^	75.7^a^	65.9^b^	1.7	<0.001
Phe	73.2^ab^	84.9^a^	66.2^b^	43.2^c^	3.8	<0.001
Thr	61.1^a^	64.9^a^	54.0^ab^	34.5^b^	4.6	0.004
Trp	77.4	85.5	65.0	51.5	8.6	0.054
Val	68.2^a^	73.5^a^	67.4^a^	43.8^b^	5.4	0.007
Dispensable amino acids						
Ala	66.8^a^	63.0^a^	63.4^a^	38.9^b^	3.6	0.001
Asp	80.6^a^	62.2^b^	61.6^b^	45.9^c^	3.0	<0.001
Cys	75.6^a^	75.2^a^	68.5^a^	51.0^b^	2.3	<0.001
Glu	86.5^a^	87.7^a^	79.9^b^	68.8^c^	1.6	<0.001
Gly	66.0^a^	55.9^a^	57.4^a^	13.1^b^	10.7	0.012
Pro	10.7	14.8	1.3	−118.5	47.2	0.063
Ser	68.8^a^	72.8^a^	61.4^a^	39.6^b^	3.7	0.001
Tyr	69.1^a^	77.7^a^	57.9^a^	−15.5^b^	10.1	0.001

Values for the coefficients of standardized ileal digestibility of crude protein and amino acids were calculated by correcting the coefficients of apparent ileal digestibility values for basal endogenous losses of crude protein and amino acids. Basal endogenous losses of amino acids (g/kg dry matter intake) were estimated using the prediction equations reported by Park et al [15]: crude protein, 19.04; Arg, 0.69; His, 0.21; Ile, 0.35; Leu, 0.57; Lys, 0.47; Met, 0.10; Phe, 0.37; Thr, 0.60; Trp, 0.13; Val, 0.51; Ala, 0.67; Asp, 0.86; Cys, 0.22; Glu, 1.08; Gly, 1.78; Pro, 5.77; Ser, 0.63; Tyr, 0.61.

a–c Least squares means without a common superscript letter within a row differ (p<0.05).

SEM, standard error of the means.

**Table 6:** Energy values (as-is basis) and digestibility in bakery byproducts fed to nursery pigs

Item	Bakery byproduct-A	Bakery byproduct-B	Bakery byproduct-C	Bakery byproduct-D	SEM	p-value
No. of observations	5	5	5	4		
GE (kcal/kg)	5,194	4,523	4,308	4,037	-	-
ATTD of GE (%)	80.2^a^	86.8^a^	68.2^b^	44.0^c^	2.3	<0.001
DE (kcal/kg)	4,175^a^	3,916^a^	2,944^b^	1,771^c^	101	<0.001
ME (kcal/kg)^1)^	4,119^a^	3,836^a^	2,870^b^	1,707^c^	101	<0.001

1) The ME was calculated using the prediction equations reported by Noblet and Perez [17]: ME (kcal/kg) = DE (kcal/kg)–0.68×crude protein (g/kg).

a–c Least squares means without a common superscript letter within a row differ (p<0.05).

SEM, standard error of the mean; GE, gross energy; ATTD, apparent total tract digestibility; DE, digestible energy; ME, metabolizable energy.

**Table 7:** Correlation coefficients among nutrient composition (% as-is), standardized ileal digestibility (SID, %) of amino acids, apparent total tract digestibility (ATTD, %) of gross energy, and digestible energy (kcal/kg as-is) in bakery byproducts fed to nursery pigs

Item	Crude protein	Ether extract	Ash	Neutral detergent fiber
Ether extract	−0.723^***^			
Ash	−0.305	−0.424		
Neutral detergent fiber	−0.032	−0.668^**^	0.942^***^	
SID of crude protein	0.004	0.495^*^	−0.639^**^	−0.718^***^
SID of Arg	0.045	0.583^**^	−0.810^***^	−0.890^***^
SID of His	0.248	0.421	−0.888^***^	−0.875^***^
SID of Ile	0.286	0.373	−0.912^***^	−0.848^***^
SID of Leu	0.194	0.328	−0.750^***^	−0.684^**^
SID of Lys	−0.649^**^	0.727^***^	−0.052	−0.348
SID of Met	0.164	0.471^*^	−0.870	−0.859^***^
SID of Phe	0.349	0.307	−0.896^***^	−0.820^***^
SID of Thr	0.196	0.378	−0.777^***^	−0.758^***^
SID of Trp	0.154	0.241	−0.569^*^	−0.516^*^
SID of Val	0.268	0.281	−0.710^***^	−0.695^**^
ATTD of gross energy	0.229	0.482^*^	−0.976^***^	−0.944^***^
Digestible energy	−0.036	0.693^**^	−0.928^***^	−0.965^***^

n = 19.

* p<0.05;

** p< 0.01;

*** p<0.001.

**Table 8:** Prediction equations for standardized ileal digestibility (SID) of crude protein and amino acids based on neutral detergent fiber (NDF) in bakery byproducts fed to nursery pigs

Item	Regression coefficient parameter		Statistical parameter		

	Intercept	NDF (% as-is)	RMSE	r^2^	p-value
SID of crude protein (%)	86.6	−0.78	8.57	0.52	0.001
SID of Arg (%)	92.1	−1.08	6.30	0.79	<0.001
SID of His (%)	81.4	−0.99	6.24	0.77	<0.001
SID of Ile (%)	84.7	−0.78	5.57	0.72	<0.001
SID of Leu (%)	83.5	−0.61	14.09	0.47	0.001
SID of Lys (%)	66.9	−0.46	14.09	0.12	0.145
SID of Met (%)	86.7	−0.54	3.66	0.74	<0.001
SID of Phe (%)	91.4	−1.24	9.82	0.67	<0.001
SID of Thr (%)	73.4	−1.00	9.83	0.57	<0.001
SID of Trp (%)	89.6	−0.98	18.54	0.27	0.024
SID of Val (%)	82.4	−0.98	11.54	0.48	0.001

The equations were developed using 19 observations.

RMSE, root mean square error.

**Table 9:** Prediction equations for apparent total tract digestibility (ATTD) of gross energy (GE) and digestible energy based on neutral detergent fiber (NDF) in bakery byproducts fed to nursery pigs

Item	Regression coefficient parameter		Statistical parameter		

	Intercept	NDF (% as-is)	RMSE	r^2^	p-value
ATTD of GE (%)	97.1	−1.38	5.46	0.89	<0.001
Digestible energy (kcal/kg as-is)	4,819	−81.96	253	0.93	<0.001

The equations were developed using 19 observations.

RMSE, root mean square error.

## Data Availability

Upon reasonable request, the datasets of this study can be available from the corresponding author.
